# Metabolic costs of submerged activity in three species of Arctic seals

**DOI:** 10.1093/conphys/coag017

**Published:** 2026-04-04

**Authors:** Madeline Meranda, Nicole M Thometz, David A S Rosen, Colleen Reichmuth

**Affiliations:** Department of Ocean Sciences, University of California Santa Cruz, 115 McAllister Way, Santa Cruz, CA 95060, USA; Alaska SeaLife Center, 301 Railway Avenue, Seward, AK 99664, USA; Department of Biology, University of San Francisco, 2130 Fulton Street, San Francisco, CA 94117, USA; Institute of Marine Sciences, University of California Santa Cruz, 115 McAllister Way, Santa Cruz, CA 95060, USA; Marine Mammal Research Unit, Institute for the Oceans and Fisheries, The University of British Columbia, 2202 Main Mall, Vancouver, BC V6T 1Z4, Canada; Alaska SeaLife Center, 301 Railway Avenue, Seward, AK 99664, USA; Institute of Marine Sciences, University of California Santa Cruz, 115 McAllister Way, Santa Cruz, CA 95060, USA

**Keywords:** Bearded seal, cost of transport, dive response, energetics, marine mammal, metabolism, respirometry, ringed seal, spotted seal, swimming

## Abstract

Arctic seals live in dynamic environments characterized by the seasonal advancement and retreat of sea ice. These amphibious marine mammals rely on sea ice as a haul-out substrate for rest and key life-history events, but they spend the majority of their time in the water. Current and predicted sea ice loss highlights the importance of estimating the costs of in-water activities when modelling the energy budgets of free-ranging seals under changing conditions. Yet, activity-specific costs are not available for many affected species. We used open-flow respirometry to measure and compare resting metabolic rates with the energetic costs of submerged diving and swimming in spotted (*Phoca largha*; *n* = 3), ringed (*Pusa hispida*; *n* = 1) and bearded seals (*Erignathus barbatus*; *n* = 1). Individuals were trained to voluntarily complete a sustained stationary breath hold under water or a continuous submerged swim before surfacing in a metabolic dome to measure rate of oxygen consumption. Metabolic rates decreased 11–24% relative to resting metabolic rate for the spotted and ringed seals while diving for 3, 5, or 7 min and did not change with increasing duration. The bearded seal did not show a similar decrease in diving metabolism. All individuals exhibited notable energetic increases to support exercise underwater while swimming at preferred speeds for 2–3 min. Metabolic costs were 243% and 114% above resting levels for spotted and ringed seals, but only 60% greater for the bearded seal. These results reveal the conflicting physiological responses of metabolic suppression while submerged (i.e. dive response) with the oxygen requirements of active swimming (i.e. exercise response) in Arctic seals and highlight the unique physiology of the bearded seal. The cost of submerged activities can now be considered in quantitative models of ice seal energy budgets, improving understanding of how physiological differences influence species-specific tolerance or vulnerability to rapidly changing Arctic conditions.

## Abbreviations

RMR: resting metabolic rate
COT: cost of transport
O_2_: rate of oxygen consumption

## Introduction

Arctic seals are uniquely adapted to occupy cold polar waters while relying on sea ice as a haul-out substrate to rest and complete physiologically taxing life-history processes. Consequently, the seasonal movements of Arctic seals tend to follow the changing boundaries of sea ice as it recedes and advances annually (Quakenbush *et al.*, 2020; Von Duyke *et al.*, 2020; Ogloff *et al.*, 2021). Human-induced climate change has caused the year-to-year persistence of sea ice to decline dramatically in both extent and quality, and extreme loss will continue (>12% per year; NASA, 2025) as the Arctic warms at an accelerated rate (Alekseev *et al.*, 2015; Laidre *et al.*, 2015; Rantanen *et al.*, 2022). Because the movement patterns and key life-history events of Arctic seals are entrained to historically predictable seasonal changes in sea ice extent, the ongoing loss of sea-ice habitat will alter the amount of time seals spend in the water, the extent of their movements and where/when critical life-history events can occur (Hamilton *et al.*, 2015, 2016; Laidre *et al.*, 2015; Von Duyke *et al.*, 2020). These behavioural changes will have significant energetic consequences for individual seals, as well as cascading biological and ecological consequences throughout Arctic and sub-Arctic ecosystems. Ice-dependent seals play important trophic roles within their respective habitats, both as high-level consumers of fish and invertebrates, and prey for apex predators (Boveng *et al.*, 2024). Further, they are essential subsistence resources for indigenous coastal communities that are especially vulnerable to rapidly changing environmental conditions (Huntington *et al.*, 2020). Given these factors, there is an immediate need to evaluate the behavioural and energetic consequences of sea ice loss in ice-dependent seals.

Bioenergetic models are powerful tools for quantifying the energetic impact of behavioural changes within a species as they can be used to determine both individual- and population-level effects of disturbances (Pirotta *et al.*, 2018), including acute and sustained changes to environmental conditions. However, effective models require accurate empirical measures of the metabolic costs of relevant behaviours in target species (McHuron *et al.*, 2022), which are difficult to obtain from free-ranging Arctic seals. There are key differences in the behaviour, morphology and physiology of different ice-associated seals, documented by traditional ecological knowledge and quantitative scientific studies (e.g. Laidre *et al.*, 2008; Gryba *et al.*, 2021; Rosen *et al.*, 2021; Thometz *et al.*, 2021; Tengler *et al.*, 2024). These differences will ultimately affect species-specific energetic expenditures and capacity to respond to changing environmental conditions (Crocker and McDonald, 2022).

Here, we focus on three species found within the Beaufort, Chukchi and Bering Seas of Alaska: spotted (*Phoca largha*), ringed (*Pusa hispida*) and bearded seals (*Erignathus barbatus*). Both ringed and bearded seals have a circumpolar distribution, while the distribution of spotted seals is limited to the North Pacific basin (Burns, 1970). Ringed seals are the smallest of this group, ranging in size from 30 to 90 kg as adults (Ferguson *et al.*, 2018; Von Duyke *et al.*, 2020). They excavate subnivean lairs in stable fast ice to use as seasonal shelters for resting, pupping and nursing (Smith and Stirling, 1975; Kunnasranta *et al.*, 2001). Ringed seals forage pelagically and dive for 3–10 min at a time in pursuit of fish and zooplankton (Gjertz *et al.*, 2000a; Harwood *et al.*, 2015; Crawford *et al.*, 2019; Ogloff *et al.*, 2021). Outside of the annual moulting period, ringed seals spend nearly 70% of their time diving (Von Duyke *et al.*, 2020). Bearded seals are much larger, with an adult body mass range of 250–360 kg (Burns and Frost, 1979), and are usually found within relatively dense broken pack ice (Burns, 1970; Bengston *et al.*, 2005). They forage almost exclusively on benthic species while typically diving for intervals of 2–10 min (Lydersen *et al.*, 1996; Gjertz *et al.*, 2000b; Hamilton *et al.*, 2018; Olnes *et al.*, 2020). Bearded seals spend an estimated 62–74% of their time diving (Hamilton *et al.*, 2018; Olnes *et al.*, 2020). Spotted seals are intermediate in size, ranging from 65–115 kg as adults (Burns, 2002). They are found at relatively lower latitudes and more commonly around the margins of seasonally shifting pack ice, whether in shallow coastal areas or in offshore waters over the continental shelf (Lowry *et al.*, 2000; Quakenbush *et al.*, 2020; Olnes *et al.*, 2023). Reports from satellite-tagged individual spotted seals indicate average dive times of <5 min while transiting or foraging for schooling fish and other species (Cordes *et al.*, 2017; Quakenbush *et al.*, 2020).

Spotted, ringed and bearded seals also exhibit distinct baseline energy requirements, with interspecific differences in resting metabolism reflecting variation in body size and physiological responses to key life-history events (Ashwell-Erickson, 1981; Ochoa-Acuña *et al.*, 2009; Thometz *et al.*, 2021, 2023). For example, ice seals display species-specific changes in resting metabolism associated with the annual moult (Thometz *et al.*, 2021, 2023). Although resting metabolism has been determined for these species while in water and when hauled-out (Thometz *et al.*, 2023), we lack the activity-specific metabolic data that are necessary to inform estimates of total energy expenditure for ice seal species, particularly measurements for routine underwater behaviour. Quantifying the metabolic cost of specific behaviours is inherently difficult because it requires linking fine-scale behavioural states to simultaneous measurements of oxygen consumption or validated energetic proxies *in situ*—an approach that is logistically infeasible for free-ranging seals in remote Arctic systems.

Studies conducted with other pinniped species in controlled laboratory settings with individuals trained to perform stationary dives have revealed reduced oxygen consumption relative to rest, attributable to the dive response (e.g. Hurley and Costa, 2001; Hastie *et al.*, 2007; Borque-Espinosa *et al.*, 2021; John *et al.*, 2021). In contrast, studies conducted with trained individuals in swim flumes have documented increased metabolic costs incurred over a range of swim speeds (e.g. Davis *et al.*, 1985; Williams *et al.*, 1991). Results from studies focused on submerged swimming behaviours (comparable to behaviour in free-ranging seals) have been associated with variable energetic responses across studies, species and age classes (e.g. Sparling and Fedak, 2004; Borque-Espinosa *et al.*, 2021; John *et al.*, 2021; Rosen, 2021). These differential results are likely driven by species- and context-specific resolutions to the conflicting needs of oxygen conservation while breath-hold diving (i.e. dive response) and oxygen use while actively moving under water (i.e. exercise response) (Davis and Williams, 2012; Noren *et al.*, 2012). Thus, metabolic data for diving and submerged swimming behaviours in Arctic seals are needed to inform our understanding of their baseline physiology, as well as parameterize robust bioenergetic models that can be used to predict energetic implications of behavioural change.

The main objectives for this study were to measure, report and compare energetic costs associated with core underwater activities in spotted, ringed and bearded seals. We used open-flow respirometry to measure the metabolic cost of three well-defined behavioural states: resting at the water’s surface, stationary diving and sustained submerged swimming. We collected these data in near-optimal laboratory conditions with five non-reproductive adult seals trained to perform sustained behavioural tasks in a relaxed manner and cooperate in repeated metabolic measurements. Resting measurements at the surface provided reference values with which to quantify the relative magnitudes of the dive response and exercise response in each individual. Stationary diving measurements were used to evaluate the dive response independent of the effects of exercise (i.e. swimming) during submersion. Given documented differences in routine diving behaviour and baseline energy demands, we expected to observe species-specific energy savings associated with the dive response. Submerged swimming measurements allowed us to document the cost of active swimming at known speeds as well as quantify the cost of transport (COT) for each individual. Together, these data fill important gaps in our understanding of the physiology of ice-dependent seals and will support ongoing efforts to evaluate the consequences of sea ice loss on these species.

## Materials and methods

### Subjects

Three spotted seals and one ringed seal at the Alaska SeaLife Center in Seward, Alaska (60.0999° N, 149.4410° W), and one bearded seal at Long Marine Laboratory in Santa Cruz, CA, USA (36.9497° N, 122.0656° W), participated in voluntary metabolic data collection for this study from June 2021 to January 2023. These were sexually mature adult males, with the exception of one adult female spotted seal (Table 1). At both facilities, seals were housed in outdoor, natural saltwater pools and surrounding enclosures for which ambient air and water temperatures were recorded hourly (TidBit v2, Onset Computer Corporation, Bourne, MA, USA). The range of average daily water temperatures by facility was 4.2–10.2°C (AK) and 8.3–15.3°C (CA) (Supplementary Data), temperatures commonly observed in Arctic and sub-Arctic waters and assumed to be within each species’ thermoneutral zone (Timmermans and Labe, 2025). Body mass was measured at least once per week with a calibrated platform scale. Seals were fed freshly thawed fish and squid according to established motivational criteria to maintain optimal health and allow for naturalistic seasonal fluctuations (Rosen *et al.*, 2021); their diets were not constrained for experimental purposes. Food intake as mass of food (kg) and total calories (kcal) was recorded daily for each individual.

**Table 1:** Subject data for five reproductively mature seals during stationary diving and submerged swimming study periods including location [Alaska SeaLife Center (AK) or Long Marine Laboratory (CA)]; subject data and environmental parameters for each sampling event are provided in Table S1

**Subjects**				**Stationary diving**			**Submerged swimming**		
**Species**	**Individual**	**Sex**	**Site**	**Age (years)**	**Mass (kg)**	**Study period**	**Age (years)**	**Mass (kg)**	**Study period**
Spotted seal	Tunu	M	AK	11	73.0–76.9	Oct 2021–Apr 2022	12	90.4–99.0	Oct 2022–Dec 2022
	Kunik	M	AK	6	71.2–81.7	Oct 2021–Feb 2022	7	99.3–102.2	Oct 2022–Jan 2023
	Sura	F	AK	7	67.0–70.1	Oct 2021–Feb 2022	8	83.4–92.6	Sept 2022–Jan 2023
Ringed seal	Pimniq	M	AK	7	40.7–42.1	Oct 2021–Feb 2022	8	42.8–45.9	Sept 2022–Jan 2023
Bearded seal	Noatak	M	CA	6	180.3–182.7	Jun 2021–Feb 2022	7	181.0–182.0	Mar 2022–Jun 2022

Previous work with these individuals highlights that spotted seals and ringed seals have increased metabolic rates during the annual moult (Thometz *et al.*, 2021). Therefore, data collection was limited to two non-moulting periods within a 15-month span for these species (Table 1). Bearded seals maintain similar metabolic rates during moulting and non-moulting periods, so data collection with this individual was not limited to non-moulting periods and occurred in two phases over 9 months (Table 1).

### Behavioural conditioning

Seals were specially trained to participate in research activities. Prior to this study, incremental operant conditioning was used to train each individual to float at the water’s surface beneath a metabolic dome. Seals maintained a resting behavioural state for a set interval before receiving a large, predictable fish reward after the trial was complete, corresponding to 30–40% of daily diet. This strategy involved gradually increasing the duration of calm, relaxed behaviour with minimal movement under the dome to prepare for the extended durations required for measuring resting metabolic rate (RMR). Routine (daily) training duration ranged from 4 to 10 min depending on species. During less frequent data collection trials, this resting interval was extended to 8–18 min. Prior to all trials, seals were fasted overnight (15+ h) to ensure a post-absorptive state and did not consume any food until the trial was complete. This conditioning approach had been used to successfully measure RMR with these individuals for at least 4 years while cooperating in similar research (Thometz *et al.*, 2021, 2023).

The seals additionally learned to perform a sustained interval of either stationary diving (i.e. near motionless behaviour at the bottom of a pool) or submerged swimming (i.e. active swimming at depth along the perimeter of a pool) on one breath hold. Selective shaping was used to establish standardized behavioural criteria, and the duration of the activity was gradually increased to target intervals (described below). Using contingency training rather than food reinforcement, each seal then learned to complete a behavioural sequence: an interval of rest up to 3 min at the surface of the water before being cued to complete either the diving or swimming activity, followed immediately by the sustained period of rest beneath the dome for metabolic data collection (Supplementary Video). Each complete behavioural sequence lasted 10–18 min and was followed by the same predictable food reward used in the rest-only condition.

Animal protocols were reviewed and approved by the Institutional Animal Care and Use Committee (IACUC) at the University of California Santa Cruz with cooperation from the IACUC at the Alaska SeaLife Center. Research permission was granted by the United States National Marine Fisheries Service (marine mammal research permits 18902, 23554) with expressed support from the Ice Seal Committee, a tribally authorized Alaska Native co-management organization.

### Respirometry

Metabolic measurements were collected using open-flow respirometry methods similar to those reported in Thometz *et al.* (2021). Briefly, a Sable Systems mass-flow controller (FlowKit Mass Flow Generator FK-500-1, Las Vegas, NV, USA) pulled air through a 150-l acrylic dome at a known flow rate between 150 and 430 l min^−1^. Flow rates were pre-determined for each trial based on animal mass and the specific activity to be performed to ensure oxygen levels remained at or above 20.1% during trials. Subsamples of air were pulled through a field metabolic system (FMS-1701-02; Sable Systems, Las Vegas, NV, USA) where air was dried (Drierite, W. A. Hammond Drierite, Xenia, OH, USA) and scrubbed of carbon dioxide (Sodasorb, Smiths Medical Inc., Minneapolis, MN, USA) prior to entering an oxygen analyser. Oxygen concentration during trials was recorded on a laptop computer using Expedata software (Sable Systems, Las Vegas, NV, USA) at a rate of one sample per second. Relative humidity and air temperature in the dome were measured with a handheld Vaisala HM40 sensor (Vantaa, Finland). Oxygen concentration measurements were corrected to standard temperature and pressure and converted to rates of oxygen consumption (O_2_) using standard equations (Withers, 1977). Baseline ambient oxygen levels were collected before and after every trial to account for any system drift. Metabolic systems at each facility were routinely tested for accuracy and leaks using 100% nitrogen gas following standard procedures (Fedak *et al.*, 1981; Davis *et al.*, 1985).

RMR was determined for each session by averaging across the lowest and most stable period of oxygen consumption, with this equilibrium period lasting at least 4 min. For diving and swimming trials, a minimum equilibrium interval of 2 min was analysed to calculate RMR following recovery from the activity. The recovery period was identified as the start of respiration in the dome until the start of equilibrium. Energy costs attributable to diving or swimming activities were calculated as the post-activity increase in O_2_ above resting levels by subtracting O_2_ during rest from O_2_ during the recovery period, while accounting for the time spent performing the activity and time to recovery.

### Stationary diving study period

To measure O_2_ during stationary diving, seals were trained to hold their breath for a predetermined amount of time in a fixed position at the bottom of a 2-m deep pool with minimal movement (Supplementary Video). To achieve these behavioural criteria, the bearded and spotted seals rested at the bottom of a pool while the more positively buoyant ringed seal rested beneath a submerged barrier. Following the prescribed dive duration within these seals’ typical diving durations, seals were cued to surface beneath the metabolic dome; a trial was aborted if the seal breathed outside of the dome before or during the recovery interval. Seals performed diving intervals of 3, 5 or 7 min, where five trials were completed at the shortest interval before increasing in duration. These intervals reflect typical dive durations exhibited in the wild and allowed us to investigate the possible role of dive duration on the extent of the dive response (e.g. Crawford *et al.*, 2019; Olnes *et al.*, 2020; Quakenbush *et al.*, 2020). Data collection occurred from October 2021 to April 2022 with the ringed and spotted seals, and from June 2021 to February 2022 with the bearded seal (Table 1). Seals participated in trials to measure O_2_ during rest only (described above) throughout this study period for at least five RMR trials to ensure that activity-specific comparisons would be anchored to appropriate reference values. Each seal later revisited the 3-min dive condition (*n* = 3–5) to assess whether any training effect had occurred.

### Submerged swimming study period

To measure O_2_ during submerged swimming, seals were trained to continuously swim underwater for 2–3 min immediately before surfacing beneath the dome (Supplementary Video). Seals swam at a consistent preferred pace while following the perimeter of a 15.6-m circular pool (AK) or a 23-m circular pool (CA). Swim speed was shaped by reinforcement training to achieve a similar time per lap without long glide intervals. Swim duration was set at 2–3 min to be similar to the lowest stationary diving interval and represent typical dive durations for these species. Data collection occurred from October 2022 to January 2023 with the ringed and spotted seals for a total of six swimming trials each, and from March 2022 to June 2022 with the bearded seal for a total of five trials (Table 1). Seals participated in at least five RMR trials during the same interval of time as the submerged swimming study period to again ensure that activity-specific comparisons would be anchored to appropriate reference values.

During each swimming trial, overhead video footage captured swim path and seal behaviour (GoPro 8, San Mateo, CA, USA; Supplementary Video). Trial video was first reviewed to produce a scaled ‘map’ of the seal’s swim path, and then ImageJ software (National Institutes of Health, Rasband, WI, USA) was used to measure the distance travelled. We used the total swim distance (m) and time interval (s) to calculate per-lap and overall swim speed (m s^−1^) for each trial.

### Analysis

Metabolic rates for each seal in each testing condition are reported as means ± SD for both absolute O_2_ (l O_2_ min^−1^) and mass-specific O_2_ (ml O_2_ min^−1^ kg^−1^). RMRs for each seal were compared across the two study periods using Welch’s two-tailed, unpaired *t* test and then combined. Within-species comparisons for the three spotted seals were evaluated using a one-way ANOVA with Tukey HSD.

Stationary diving conditions (3, 5, 7 min) were evaluated for each seal to reveal potential differences in metabolic rate by dive duration using a one-way ANOVA with Tukey HSD *post hoc* comparisons. Measures of metabolic costs during stationary diving were subsequently pooled for each seal as no systematic differences with increasing duration were found for any individual. Post-study repeated trials for the 3-min dive condition were evaluated within individuals to detect possible practice or training effects, but these data were not included in subsequent formal analyses. Metabolic costs attributable to submerged swimming were reported for each seal. We also calculated COT (ml O_2_ kg^−1^ m^−1^), defined as the energy required to transport a unit of body mass a set unit of distance, for each trial by dividing the mass-specific swimming metabolic rate by swim speed.

Given limited samples of one to three individuals per species, we were unable to fit a single linear mixed effects model to examine the interaction between species and activity type. Therefore, to evaluate the presence of a dive response by species, we compared mass-specific metabolism between the resting condition and the stationary diving condition using Welch’s two-tailed, unpaired *t* tests. Next, we assessed differences between resting, stationary diving and submerged swimming behaviours for each species with one-way ANOVA and Tukey HSD *post hoc* comparisons. For spotted seal statistical analyses, data from all three individuals were pooled. Statistical tests were run using GraphPad Prism v. 9.5.1. with alpha level set at 0.05.

## Results

The seals completed 176 trials meeting strict behavioural criteria for each activity type. These included at least 10 resting state, 15 stationary diving and 5–6 submerged swimming trials per individual (Supplementary Data).

RMR values associated with both study periods (i.e. stationary diving and submerged swimming trials) were similar within individuals (Welch’s *t* test, *P* > 0.05). This confirmed that for each seal, RMR values from both study periods could be combined and used for comparisons with activity-specific metabolic rates. Stationary diving metabolic rates did not change with increasing duration (one-way ANOVA with Tukey HSD, *P* > 0.05; Fig. S1), and thus data from the 3-, 5- and 7-min diving conditions were pooled by individual for subsequent analyses. Repeating several trial replicates at the 3-min duration with all individuals following the study period did not reveal a training effect, which would have been indicated by an overall decrease in observed diving costs relative to initial measurements.

### Spotted seal

**Figure 1:**
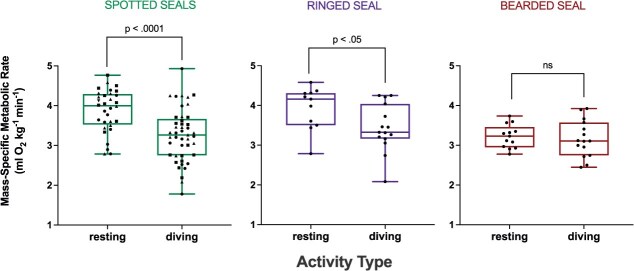
Mass-specific metabolic rates measured in each species during resting and stationary diving. Boxes extend from median to the 25th and 75th percentiles, and whiskers extend to the min and max points. Statistical results from the pairwise comparisons (Welch’s *t* test) are noted above brackets for the spotted seals (pooled; *n* = 3), ringed seal (*n* = 1) and bearded seal (*n* = 1). Symbols represent data from individual trials; data for each spotted seal are represented by different symbols (*Tunu* = ■, *Kunik* = 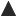, *Sura* = 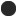).

**Figure 2:**
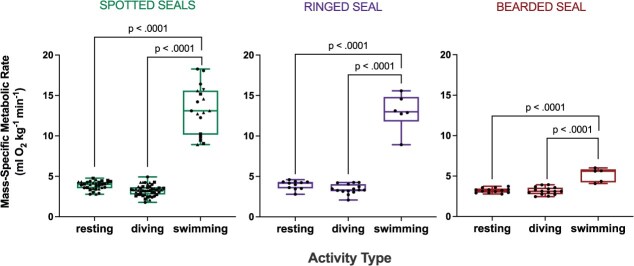
Mass-specific metabolic rates measured in each species during three activity types (resting state, stationary dive and submerged swim). Boxes extend from median to the 25th and 75th percentiles, and whiskers extend to the min and max points. Statistical results from the pairwise comparisons (one-way ANOVA) are noted above brackets for the spotted seals (pooled; *n* = 3), ringed seal (*n* = 1) and bearded seal (*n* = 1). Symbols represent data from individual trials; data for each spotted seal are represented by different symbols (*Tunu* = ■, *Kunik* = 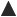, *Sura* = 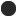).

The spotted seals’ mean RMR while at the water’s surface was 0.32 ± 0.05 l O_2_ min^−1^ on an absolute basis, with a mass-specific RMR of 3.88 ± 0.53 ml O_2_ min^−1^ kg^−1^ (Table 2). Mass-specific RMR was similar among these mature individuals (one-way ANOVA, *P* > 0.05). On a mass-specific basis, the metabolic rate associated with stationary diving for the spotted seals was 3.26 ± 0.80 ml O_2_ min^−1^ kg^−1^, 16% lower than the RMR value (Welch’s *t* test, *P* < 0.001; Fig. 1 and Table 2). During submerged swimming trials, the spotted seals swam 183–250 m each trial at a stable preferred speed of 1.4 ± 0.12 m s^−1^. Metabolic rate while submerged swimming was 13.31 ± 2.96 ml O_2_ min^−1^ kg^−1^, with an associated COT of 0.17 ± 0.03 ml O_2_ kg^−1^ m^−1^ (Table 3). Submerged swimming resulted in a marked increase in metabolic rate relative to both resting and stationary diving behaviours (one-way ANOVA, *P* < 0.001; Fig. 2). RMR and stationary diving costs were not statistically different from one another when compared with submerged swimming costs, due to the magnitude of the metabolic response associated with exercise (one-way ANOVA, *P* > 0.05). Specifically, individual metabolism increased 181–328% while submerged swimming relative to rest (Table 3).

**Table 2:** Mean ± SD absolute and mass-specific metabolic rates of each seal during resting and stationary diving trials; percentage change in metabolic rate from resting to stationary diving calculated from mass-specific values

**Subjects**		**Absolute metabolic rate (l O**_**2**_ **min**^**−1**^**)**		**Mass-specific metabolic rate (ml O**_**2**_ **kg**^**−1**^ **min**^**−1**^**)**		**% Change**
**Species**	**Individual**	**Resting**	**Diving**	**Resting**	**Diving**	**Rest–dive**
Spotted seal	Tunu	0.35 ± 0.03 (*n* = 10)	0.24 ± 0.05 (*n* = 15)	4.12 ± 0.42 (*n* = 10)	3.13 ± 0.60 (*n* = 15)	−24
	Kunik	0.34 ± 0.04 (*n* = 11)	0.26 ± 0.05 (*n* = 15)	3.93 ± 0.54 (*n* = 11)	3.44 ± 0.60 (*n* = 15)	−13
	Sura	0.28 ± 0.04 (*n* = 11)	0.22 ± 0.05 (*n* = 15)	3.60 ± 0.53 (*n* = 11)	3.21 ± 0.77 (*n* = 15)	−11
	(all)	0.32 ± 0.05 (*n* = 32)	0.24 ± 0.06 (*n* = 45)	3.88 ± 0.53 (*n* = 32)	3.26 ± 0.80 (*n* = 45)	−16
Ringed seal	Pimniq	0.19 ± 0.02 (*n* = 10)	0.15 ± 0.02 (*n* = 15)	4.52 ± 0.35 (*n* = 10)	3.59 ± 0.41 (*n* = 15)	−21
Bearded seal	Noatak	0.58 ± 0.05 (*n* = 13)	0.58 ± 0.09 (*n* = 15)	3.22 ± 0.05 (*n* = 13)	3.16 ± 0.46 (*n* = 15)	−2

**Table 3:** Mean ± SD absolute and mass-specific metabolic rates of each seal during submerged swimming with associated swim speed and COT data; percentage change in metabolic rate from resting to submerged swimming calculated from mass-specific values

**Subjects**		**Absolute metabolic rate (l O**_**2**_ **min**^**−1**^**)**	**Mass-specific metabolic rate (ml O**_**2**_ **kg**^**−1**^ **min**^**−1**^**)**	**% Change**	**Swim speed (m s** ^**−1**^**)**	**COT (ml O**_**2**_ **kg**^**−1**^ **m**^**−1**^**)**
**Species**	**Individual**			**Rest–swim**		
Spotted seal	Tunu	1.09 ± 0.28 (*n* = 6)	11.58 ± 3.02 (*n* = 6)	181	1.3 ± 0.10	0.15 ± 0.03
	Kunik	1.31 ± 0.24 (*n* = 6)	12.97 ± 2.27 (*n* = 6)	230	1.5 ± 0.02	0.17 ± 0.03
	Sura	1.39 ± 0.23 (*n* = 6)	15.39 ± 2.57 (*n* = 6)	328	1.3 ± 0.09	0.17 ± 0.02
	(all)	1.26 ± 0.27 (*n* = 18)	13.31 ± 2.96 (*n* = 18)	243	1.4 ± 0.12	0.17 ± 0.03
Ringed seal	Pimniq	0.43 ± 0.07 (*n* = 6)	9.66 ± 1.81 (*n* = 6)	114	1.2 ± 0.06	0.13 ± 0.02
Bearded seal	Noatak	0.93 ± 0.16 (*n* = 5)	5.14 ± 0.87 (*n* = 5)	60	0.8 ± 0.06	0.11 ± 0.01

### Ringed seal

The adult male ringed seal’s mean RMR while at the water’s surface was 0.19 ± 0.02 l O_2_ min^−1^ on an absolute basis, with a mass-specific RMR of 4.52 ± 0.35 ml O_2_ min^−1^ kg^−1^ (Table 2). On a mass-specific basis, metabolic rate during stationary diving was 3.59 ± 0.41 ml O_2_ min^−1^ kg^−1^, which was 21% lower than the RMR value (Welch’s *t* test, *P* < 0.05; Fig. 1 and Table 2). During submerged swimming trials, the ringed seal swam 179–215 m at a highly consistent preferred speed of 1.2 ± 0.06 m s^−1^. The submerged swimming metabolic rate was 9.66 ± 1.81 ml O_2_ min^−1^ kg^−1^, and COT was 0.13 ± 0.02 ml O_2_ kg^−1^ m^−1^ (Table 3). Similar to the spotted seals, submerged swimming was associated with a notable increase in metabolic rate relative to both resting and stationary diving behaviours (one-way ANOVA, *P* < 0.001; Fig. 2). Again, RMR and stationary diving costs were not statistically different from one another when compared with submerged swimming costs, due to the metabolic response associated with exercise (one-way ANOVA, *P* > 0.05). The ringed seal’s metabolic rate increased 114% while submerged swimming relative to rest (Table 3).

### Bearded seal

The adult male bearded seal’s mean RMR while at the water’s surface was 0.58 ± 0.05 l O_2_ min^−1^ on an absolute basis, with a mass-specific RMR of 3.22 ± 0.05 ml O_2_ min^−1^ kg^−1^ (Table 2). His metabolic rate during stationary diving was 3.16 ± 0.46 ml O_2_ min^−1^ kg^−1^, the same as his RMR measured at the water’s surface (Welch’s *t* test, *P* > 0.05, Fig. 1 and Table 2). During swimming trials, the bearded seal swam 101–122 m at a stable preferred speed of 0.8 ± 0.06 m s^−1^. Submerged swimming metabolic rate was 5.14 ± 0.87 ml O_2_ min^−1^ kg^−1^, and COT was 0.11 ± 0.01 ml O_2_ kg^−1^ m^−1^ (Table 3). Submerged swimming was associated with a significant increase in metabolic rate relative to both resting and stationary diving behaviours (*P* < 0.0001; Fig. 2). RMR and stationary diving costs were again similar when compared with submerged swimming costs (one-way ANOVA, *P* > 0.05). The bearded seal incurred a 60% increase in metabolism while submerged swimming relative to rest (Table 3).

## Discussion

Here we report key bioenergetic parameters for three ice-associated seal species. As expected, absolute baseline metabolic costs scaled with body size, being highest in the bearded seal and lowest in the ringed seal. The spotted seals and ringed seal exhibited significant metabolic depression during stationary diving relative to rest, which was comparable among individuals and indicative of a dive response (Hurley and Costa, 2001; Hastie *et al.*, 2007; Rosen *et al.*, 2017). The bearded seal did not show a similar metabolic response associated with stationary diving. Metabolic rates increased significantly during submerged swimming for all individuals and species compared to both resting and stationary diving metabolic rates. Specifically, we measured 2.8- to 4.2-fold increases in metabolism from resting to swimming for the spotted seals, a 2.2-fold increase for the ringed seal and a 1.6-fold increase for the bearded seal. The bearded seal also had the lowest COT and the slowest preferred swim speed among the ice seals in this study. Overall, the values reported here expand the limited pool of ‘gold standard’ resting and activity-specific metabolic data available for marine mammals (Noren and Rosen, 2023) and double the number of phocid seal species for which COT data are available.

### Resting metabolic rates

RMR was measured and reported to provide within-individual reference values to compare to rates of energy expenditure in other behavioural states. Although RMR data were collected throughout the duration of this study to ensure appropriate matching between resting and activity-specific measurements, each seal showed low variance (e.g. high repeatability) in RMR values. Phocid seals generally have lower baseline metabolic costs than other marine mammals when considered by body size (Williams, 2022), with the exception of sirenians that maintain the lowest baseline energetic costs (John *et al.*, 2021). This was true for the seals in this study. In fact, our seals showed some of the lowest RMR values among marine mammals measured thus far (Noren and Rosen, 2023), despite living in cold Arctic waters where thermal costs can be substantial. Although a limited number of individuals participated in this study, our RMR data align with previously published values from the same animals as well as additional individuals of these species (Thometz *et al.*, 2021, 2023).

### Stationary diving energetic costs

The majority of marine mammals have a pronounced dive response—defined by apnea, bradycardia and peripheral vasoconstriction—that facilitates oxygen conservation while diving in order to extend aerobic dive times (Ponganis, 2011). The *a priori* prediction that we would observe a depression in metabolic rate attributable to the dive response was confirmed for the spotted and ringed seals. The metabolic decrease observed during the stationary diving condition ranged from 11% to 24% below mass-specific RMR for these four individuals. In contrast, this decrease was negligible (2%) for the bearded seal, with the possible exception of the longest dive condition (see Fig. S1), suggesting a blunted or minimal dive response. Other studies that have examined metabolic changes in diving pinnipeds have documented similar decreases during submersion. When performing stationary dives, trained Pacific walruses (*Odobenus rosmarus divergens*) conducting 3-min dives exhibited a 18% decrease in mass-specific metabolism (Borque-Espinosa *et al.*, 2021), trained Steller sea lions (*Eumetopias jubatus*) performing 1- to 3-min open ocean dives displayed a 45% decline (Hastie *et al.*, 2007) and spontaneously diving (average = 6.4 min; maximum = 16 min) juvenile northern elephant seals (*Mirounga angustirostris*) showed a 26% reduction (Webb *et al.*, 1998). One study examining this phenomenon in trained Hawaiian monk seals (*Neomonachus schauislandi*) reported a 35% reduction in metabolism for an adult seal that completed 2–8 min stationary dives, but only an 8% decrease in a single juvenile (John *et al.*, 2021). Our work adds to a growing body of literature highlighting variability in the metabolic responses to diving across species.

The bearded seal’s apparent lack of a metabolic response to extended submersion is an outlier among existing marine mammal data. In pinnipeds, the dive response has most commonly been demonstrated by reductions in heart rate (Kooyman and Campbell, 1972; Williams *et al.*, 1991; Reed *et al.*, 1994; Ponganis *et al.*, 1997; Webb *et al.*, 1998). Bradycardia decreases oxygen consumption directly via lower cardiac costs and indirectly by facilitating peripheral vasoconstriction, which promotes the use of myoglobin-bound oxygen in skeletal muscle. Thus, deeper levels of bradycardia are associated with lower metabolic costs (Rosen *et al.*, 2017; Noren *et al.*, 2023). While there are few data to describe heart rate patterns during diving in these ice-associated species (see Elsner *et al.*, 1989), available evidence indicates that spotted and ringed seals exhibit routine bouts of apnea accompanied by pronounced bradycardia, even when resting on land, indicative of a substantive dive response (Jones *et al.*, 2021; Reichmuth, unpublished data). This is similar to what has been documented in other phocids (e.g. Falabella *et al.*, 1999; Kaczmarek *et al.*, 2018). In contrast, bearded seals breathe more regularly and display only brief apneas when hauled out (Jones *et al.*, 2021; Reichmuth, unpublished data), a pattern that is atypical among phocids but aligns with the negligible metabolic suppression observed during stationary diving for the bearded seal in this study.

Although there are true differences in how the dive response manifests across marine mammals, including the magnitude and timing of bradycardia, peripheral perfusion and metabolic suppression (Ponganis, 2011), some of the variability observed in available literature is likely due to methodological differences. For example, trained Steller sea lions (Hastie *et al.*, 2006) and free-ranging Weddell seals (*Leptonychotes weddellii*; Castellini *et al.*, 1992; Williams *et al.*, 2004) incur greater metabolic savings when diving to deeper depths, a variable that was held constant in our study. In John *et al.* (2021), the length of each dive for their Hawaiian monk seals was randomized across trials and unknown to their study animals; the authors acknowledged that this may have elicited a larger metabolic reduction than would have otherwise been observed if the seals could anticipate the duration of each dive trial. The stationary diving data reported here for our ice seals were obtained under relaxed, predictable conditions in which each individual was familiar with the dive duration they would be asked to perform. Thus, these data approximate ‘normal’ breath-hold conditions of free-ranging ice seals rather than maximum dive responses (see Ponganis *et al.*, 2011). Of note, our seals did not show a systematic decrease in metabolism with increasing dive duration; with the potential exception of the longest dive condition (7 min) in the bearded seal (see Fig. S1). This is in contrast to observations of trained California sea lions (*Zalophus californianus*) and Steller sea lions performing stationary dives of similar durations to our subjects (Gerlinsky *et al.*, 2013, 2014; Hurley and Costa, 2001), as well as grey (*Halichoerus grypus;*  Reed *et al.*, 1994; Sparling and Fedak, 2004) and northern elephant seals (Webb *et al.*, 1998) performing spontaneous dives of similar and longer durations in a laboratory setting. The fact that our seals did not exhibit a consistent decrease in metabolism with increasing stationary dive duration may be due to the fact that many of the studies mentioned above did not match our stationary dive criteria for direct comparison (i.e. seals were not fully stationary underwater and were not always post-absorptive) or may suggest that our dive times did not include a wide enough range to reveal this physiological phenomenon in ice seals.

### Submerged swimming energetic costs

While it is unknown how often behaviour akin to our stationary dive condition occurs in wild individuals, measuring this condition enabled us to isolate the potential energy conserving effects of the dive response from the energetic effects of the exercise response when submerged swimming. We found that sustained swimming during submersion resulted in metabolic costs that were much higher than RMR values for all individuals. The spotted and ringed seals increased oxygen consumption during swimming by 243% and 114% above resting levels, respectively, while the bearded seal’s oxygen consumption during swimming only increased by 60%. The conflicting effects of the dive response and exercise response are complex and deeply intertwined. Factors such as the physiological state of an individual, dive duration and exercise intensity, among others, influence both responses (Davis and Williams, 2012; Rosen *et al.*, 2017; Costa and Favilla, 2023). This dynamic relationship merits further exploration within and across marine mammal species.

It is unclear whether higher metabolic costs during swimming are the net result of additional energetic expenditures above the measured savings accrued from metabolic depression during submergence, or whether the activity of swimming diminishes the extent of the dive response itself. Previous studies of marine mammals have demonstrated bradycardia during activity underwater (Ponganis *et al.*, 1990; Reed *et al.*, 1994; Williams *et al.*, 2015), which Ponganis *et al.* (1997) reported as similar to bradycardia observed during trained stationary submersion in California sea lions. In other marine mammal cases, bradycardia appears modified to support increasing levels of exercise (Davis and Williams, 2012; Noren *et al.*, 2012; Williams *et al.*, 2015). While the results of our study cannot disentangle the relationship between the exercise response and the dive response in our target species, we hypothesize that the cost of exercise observed should incorporate some of the savings measured during stationary diving—due to the dive response—while still resulting in a net increase in metabolism above the resting state. Further examination of the physiological processes at work (e.g. heart rate, tissue perfusion) during submerged conditions with and without exercise would help reveal the extent to which the dive response persists during submerged activity in these species.

**Figure 3:**
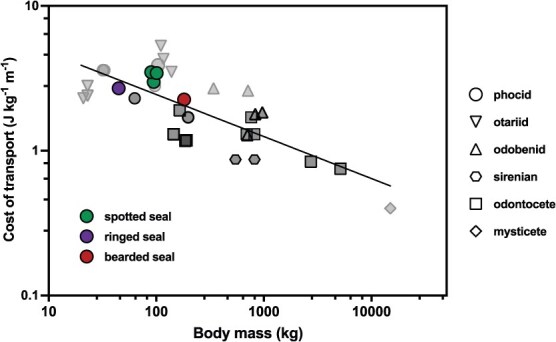
Calculated COT for spotted, ringed and bearded seals relative to body mass on a log–log scale. Comparative measurements available for other swimming marine mammals are shown in dark grey (adults) and light grey (juveniles). The allometric relationship for these data is shown by the black line (COT = 9.29 × mass^−0.29^). Phocids (grey seal *H. grypus*: Fedak, 1986; harbour seal *Phoca vitulina*: Williams *et al.*, 1991*; Davis *et al.*, 1985*; Hawaiian monk seal *Neomonachus schauinslandi*: John *et al.*, 2021), otariids (California sea lion *Z. californianus*: Williams *et al.*, 1991*; Williams, 1999; Feldkamp, 1987*; Steller sea lion *E. jubatus*: Rosen and Trites, 2002), odobenids (Pacific walrus *O. rosmarus divergens*: Rosen, 2021; Borque-Espinosa *et al.*, 2021), sirenians (West Indian manatee *Trichechus manatus*: John *et al.*, 2021), odontocetes (killer whale *Orcinus orca*: Kriete, 1995*; bottlenose dolphin *Tursiops truncatus*: Williams *et al.*, 1993; Williams, 1999; van der Hoop *et al.*, 2014*; Allen *et al.*, 2022*; John *et al.*, 2024; beluga whale *Delphinapterus leucas*: John *et al.*, 2024), mysticetes (grey whale *Eschrichtius robustus*: Sumich, 1983*). Data are displayed by individual when possible; the asterisk symbol (*) indicates when a study combined data from multiple individuals.

Freely diving marine mammals are known to behaviourally reduce costs while submerged by alternating their swimming pattern between gliding and active stroking (e.g. Williams *et al.*, 2000; Hindle *et al.*, 2010), thereby reducing the average cost of movement. This strategy may explain reports of metabolic rates during swimming that are similar or reduced relative to resting states in other pinnipeds (e.g. Castellini *et al.*, 1992; Sparling and Fedak, 2004; Fahlman *et al.*, 2008; Borque-Espinosa *et al.*, 2021) despite the additional burden of swimming intensity or distance (Hastie *et al.*, 2006; Davis and Williams, 2012). Seals swimming underwater also take advantage of the hydrodynamic benefits of full submersion, compared with swimming against additional drag at the surface of the water (Williams and Kooyman, 1985), which is clearly demonstrated in some laboratory studies of swimming metabolic rates (Davis *et al.*, 1985; Williams *et al.*, 1991; Rosen and Trites, 2002). The seals in this study swam in a pool while fully submerged (i.e. no surface drag) and consistently stroking with their hind flippers (Supplementary Video). Because of this continuous effort, the metabolic costs reported here for submerged swimming may be on the higher end of those experienced by free-ranging ice seals that may avail themselves of energy-saving behaviours. Similar to the seals in our study, significant elevations in submerged swimming costs relative to rest have also been observed in trained Hawaiian monk seals and juvenile walruses performing comparable tasks (John *et al.*, 2021; Rosen, 2021).

Although our seals were asked to swim continuously, each individual was able to swim at their preferred (i.e. self-selected) swimming speed. This was an important aspect of our study design as the energetic cost of swimming a given distance depends on velocity (Schmidt-Nielsen, 1972), and marine mammals typically swim at preferred cruising speeds that reduce overall costs (Williams, 1999). Across species, this energetic cost should decrease with body size (Schmidt-Nielsen, 1972). Given that our study focused on one to three individuals per species, we could not define species-specific optimal velocities; however, by quantifying individual preferred swim speeds, we were able to directly calculate the COT for each seal. The spotted seals had the highest COT in this study, but their values were similar to those reported for immature and adult harbour seals—their closest phocid relative—swimming at similar speeds (Davis *et al.*, 1985; Williams *et al.*, 1991; Quakenbush *et al.*, 2009). The bearded seal had the lowest COT of all study animals but also had a consistently slower velocity (0.8 m s^−1^) while swimming than the spotted seals (1.3–1.5 m s^−1^) and the ringed seal (1.2 m s^−1^). Its slower preferred swim speed is reflective of the typical low and slow nature and physiology of bearded seals (Reichmuth and Klein, 2025) and provides further support that this species has unique physiological and behavioural characteristics (e.g. metabolic patterns, moulting physiology, swimming behaviour) among Arctic phocids (Thometz *et al.*, 2021, 2023).

The data reported here doubles the number of phocid seal species for which comparative COT data are available (Fig. S2). When viewed broadly, the COT values we determined for spotted, ringed and bearded seals conform remarkably well with the available data for marine mammals generally. The allometric relationship reported here (Fig. 3; COT = 9.29 × mass^−0.29^) using all available COT data for swimming marine mammals (including data from this study) remains similar to the relationship originally defined by Williams (1999) (COT = 7.79 × mass^−0.29^). Although both allometric relationships necessarily combined adult and immature COT values, there is still notable consistency across a wide range of body sizes. Despite having the lowest COT of our target species, the bearded seal’s value falls right in line with the predicted relationship for marine mammals when body size is taken into account. The ringed and spotted seal data also conform well to predictions based on body size. Overall, this evidence supports the use of our metabolic values when modelling the energetic consequences of behavioural change in these three species.

### Conservation implications

Continued warming in the Arctic threatens to alter the routine behaviour of ice-dependent seals, including seasonal movements and haul-out patterns (Hamilton *et al.*, 2016; Breed *et al.*, 2018; Cameron *et al.*, 2018; Von Duyke *et al.*, 2020). Consequences stemming from these changes have been difficult to anticipate given that the metabolic data required to accurately quantify the energetic implications of behavioural change have been unavailable for these target species until now. We identified activity-specific metabolic differences across three seal species that are important to consider in modelling efforts and highlight the value of obtaining species-specific physiological data for diving mammals that exhibit morphological, behavioural and phylogenetic differences (Booth *et al.*, 2023). The measured RMRs reported here provide further evidence that phocid seals have lower baseline energy requirements than other marine mammals and that bearded seals exhibit particularly low mass-specific metabolic rates even among phocids. Comparison of RMR values to stationary diving costs revealed notable metabolic suppression during diving for the spotted and ringed seals, but little evidence of a metabolic reduction in the bearded seal. Finally, controlled exercise trials provided information about preferred swim speeds, the cost of submerged swimming and COT in these species. Scientists can now incorporate the metabolic cost of various surface and submerged behaviours when linking metabolic data to activity budgets derived from biologging efforts with wild individuals in catch and release programmes (e.g. Von Duyke *et al.*, 2020; London *et al.*, 2024). Most importantly, the development of robust bioenergetic and/or population models using these empirically derived data can be used to better predict species-specific consequences of rapid habitat change in Arctic and sub-Arctic environments.

## Supplementary Material

Web_Material_coag017

## Data Availability

Metabolic trial and associated subject and environmental data are available in the online supplementary material (Supplementary Data), which includes energetic costs expressed as both absolute and mass-specific values.
