# Supplementary material for: Metabolic costs of submerged activity in three species of Arctic seals
Source: Conserv Physiol. 2026 Apr 4;14(1):coag017. doi: 10.1093/conphys/coag017 (PMC13056718; doi:10.1093/conphys/coag017)
Supplement: Web_Material_coag017 [file web_material_coag017.zip › Supplementary_Figures_second_revision.pdf]

Supplementary Figures

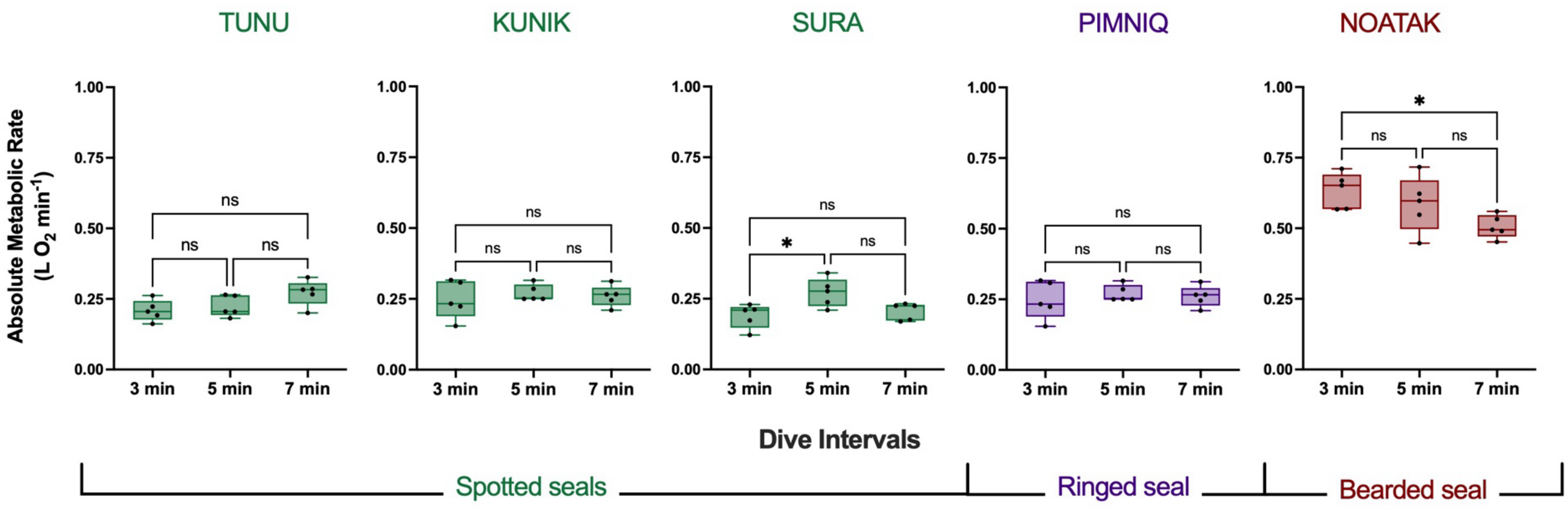

Supplementary Figure 1. Comparison of dive conditions by individual. Absolute metabolic rates were evaluated for 3 min, 5 min, and 7 min diving blocks within individual (one-way ANOVA with Tukey HSD; Supplementary Data) before collapsing the data into one "stationary diving" condition. We found no systematic change in metabolic rate between these conditions, with the possible exception of the bearded seal for the longest dive duration examined (7 min).

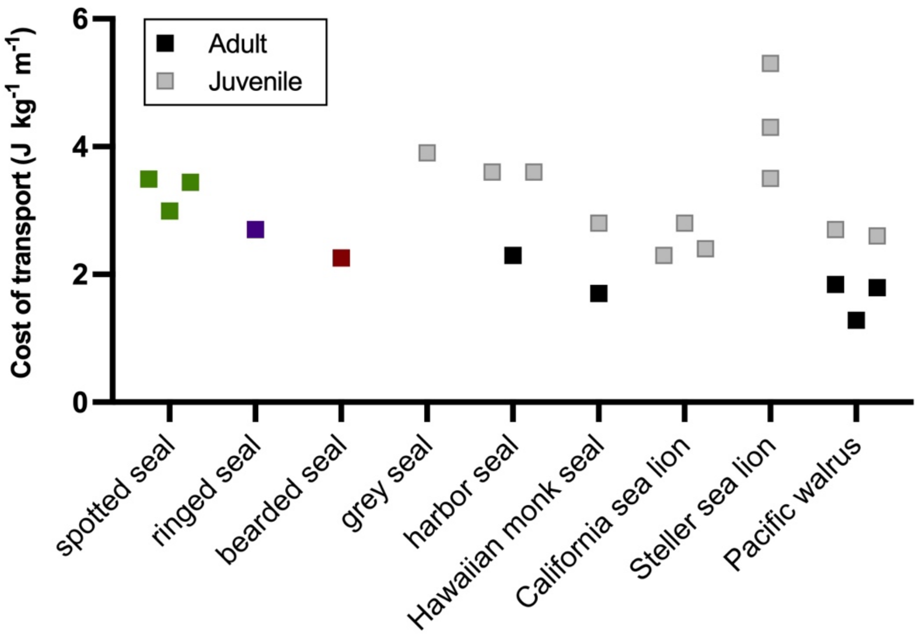

Supplementary Figure 2. Measured cost of transport for adult spotted, ringed, and bearded seals (colored, filled) relative to other pinnipeds. Comparative data points for pinnipeds represent adult (black) and juvenile (grey) individuals, unless otherwise noted. Grey seal: Fedak, 1986*; harbor seal: Williams *et al.*, 1991*; Davis *et al.*, 1985*; Hawaiian monk seal: John *et al.*, 2021; California sea lion: Williams *et al.*, 1991*; Williams, 1999; Feldkamp, 1987*; Steller sea lion: Rosen and Trites, 2002, Pacific walrus: Rosen, 2021; Borque-Espinosa *et al.*, 2021). Data are displayed Data are displayed by individual when possible; * indicates when a study combined data from multiple individuals.
